# Effective image compression using transformer and residual network for balanced handling of high and low-frequency information

**DOI:** 10.1371/journal.pone.0333376

**Published:** 2025-10-03

**Authors:** Jianhua Hu, Guixiang Luo, Xiangfei Feng, Zhanjiang Yuan, Jiahui Yang, Wei Nie

**Affiliations:** 1 Computer Engineering Technical College, Guangdong Polytechnic of Science and Technology, Zhuhai, Guangdong, China; 2 College of Computer Science, Guangdong Polytechnic Normal University, Guangzhou, Guangdong, China; Menoufia University, EGYPT

## Abstract

Image compression has made significant progress through end-to-end deep-learning approaches in recent years. The Transformer network, coupled with self-attention mechanisms, efficiently captures high-frequency features during image compression. However, the low-frequency information in the image cannot be obtained well through the Transformer network. To address this issue, the paper introduces a novel end-to-end autoencoder architecture for image compression based on the transformer and residual network. This method, called Transformer and Residual Network (TRN), offers a comprehensive solution for efficient image compression, capturing essential image content while effectively reducing data size. The TRN employs a dual network, comprising a self-attention pathway and a residual network, intricately designed as a high-low-frequency mixer. This dual-network can preserve both high and low-frequency features during image compression. The end-to-end training of this model employs rate-distortion optimization (RDO methods). Experimental results demonstrate that the proposed TRN method outperforms the latest deep learning-based image compression methods, achieving an impressive 8.32% BD-rate (bit-rate distortion performance) improvement on the CLIC dataset. In comparison to traditional methods like JPEG, the proposed achieves a remarkable BD-rate improvement of 70.35% on the CLIC dataset.

## 1. Introduction

With the advent of 5G technology, there has been explosive growth in image and video data in our daily lives. Image compression is becoming increasingly critical in this era of big data, as it effectively reduces data storage demands and facilitates efficient transmission. In recent years, deep learning technologies have demonstrated superior performance compared to traditional image compression methods such as JPEG and BPG [1]. Among deep learning architectures, the Variational Autoencoder (VAE) is frequently employed, involving two key components for optimization. **The first component** focuses on transforming the original image into a lower-dimensional latent space representation using non-linear transformations. Various neural network architectures are utilized for this purpose, including Recurrent Neural Networks (RNNs), Convolutional Neural Networks (CNNs), and Transformer networks. For example, Toderici et al. [2] pioneered an RNN-based image compression model, while Ballé et al. [3] introduced an innovative compression approach using CNNs. More recently, hybrid models combining different architectural strengths have shown great promise [4]. Lu et al. [5] made significant contributions with a unique Transformer network-based method. **The second component** centers on estimating data probability distributions using neural networks. This estimation aims to create a more accurate entropy coding model, thereby enhancing entropy coding performance and reducing the bit rate of the coded stream for latent space data transformation. For instance, Ballé et al. [6] introduced the ‘scale hyperprior’ to improve entropy coding probability estimation.

The Transformer network model, a deep learning architecture based on self-attention mechanisms, has been increasingly adopted for image compression. Researchers [5,7] have adopted the Transformer to improve the performance of autoencoder-based image compression, achieving notable results. However, these methods often fall short in capturing low-frequency features, leading to an ineffective extraction of flat region characteristics within the image information. This limitation stems from the Transformer’s inherent design, which prioritizes long-range dependencies and high-frequency details, potentially overlooking local and smooth regions.

To tackle this issue, this paper presents a novel approach that integrates global modeling based on Transformers and local detail capture. The proposed architecture features a parallel image compression network, incorporating both a self-attention path and a CNN residual network as a high-low frequency mixer. This configuration enables the simultaneous modeling of discriminative information across both extensive regions and local areas. This hybrid approach aims to overcome the limitations of Transformer-only architectures by providing a more balanced representation of image features, encompassing both high and low-frequency components. This work significantly extends our preliminary findings presented in [8], introducing a more sophisticated network architecture, comprehensive ablation studies, and a more comprehensive experimental analysis.

The main contributions of this paper are as follows:

1. A novel Transformer and CNN residual Network (TRN) is proposed in a unified manner. This architecture effectively extracts features from flat regions in image data, enabling the incorporation of both high-frequency and low-frequency information for comprehensive feature representation in image compression.
2. Introduced a novel network structure that runs CNN residual network and Transformer in parallel, addressing the limitation of Transformer in capturing local features. This network demonstrates efficient extraction of image compression features and achieves superior compression performance by reducing the network’s structural levels.
3. Experiments were conducted on the CLIC dataset, the proposed method exhibited outstanding performance in terms of bitrate-distortion characteristics and visual quality, surpassing current mainstream solutions.

## 2. Related work

Current research in image compression increasingly integrates neural networks to improve coding efficiency. The primary approaches can be broadly categorized into CNN-based, Transformer-based, and hybrid methods, each with distinct advantages and limitations.

**CNN-Based Networks applied in image compression**: Convolutional Neural Networks (CNNs) have long been the cornerstone of deep learning-based image compression. Their inherent inductive bias for locality and spatial hierarchy makes them highly effective at extracting local features and textures. Early works by Ballé et al. [3,6] established a foundational VAE framework with CNNs for both the main and hyperpriors, demonstrating significant gains over traditional codecs. Subsequent research focused on improving network architecture, such as incorporating residual blocks to ease the training of deeper networks and enhance feature representation [9,10]. For instance, Cheng et al. [10] utilized residual learning with attention modules to improve rate-distortion performance. However, a primary limitation of pure CNN-based methods is their restricted receptive field, which makes it challenging to model long-range dependencies and global context within an image effectively. This can result in suboptimal compression in images with large, uniform areas or complex global structures.

**Transformer-Based Networks applied in image compression**: The Transformer’s self-attention mechanism, initially developed for natural language processing [11], has demonstrated remarkable effectiveness in various visual tasks [12,13] by capturing global dependencies. Its application to image compression has led to significant performance improvements [7]. For instance, the work on TinyLIC [14] showcased the potential of a lightweight Transformer-based model for efficient image compression. These models excel at modeling the global context of an image, dynamically prioritizing important regions and allocating bits more effectively. However, Transformer-based methods are not without drawbacks. They often require large amounts of training data and can be computationally intensive. More importantly, their standard formulation may neglect fine-grained local details and low-frequency information, which are crucial for maintaining high fidelity in smooth regions of an image. This can lead to the introduction of subtle artifacts or a loss of texture.

**Hybrid and Advanced Networks applied in image compression**: To address the complementary strengths and weaknesses of CNNs and Transformers, a growing body of research has focused on developing hybrid architectures. These models aim to combine the local feature extraction power of CNNs with the global context modeling capabilities of Transformers. Liu et al. [4], for example, proposed a dual-domain network based on hybrid convolution for hyperspectral image super-resolution, demonstrating the power of fusing different network types. In a similar spirit, our work designs a parallel structure to explicitly handle both high- and low-frequency information. Furthermore, the broader field of computer vision continues to push the boundaries of model design with advanced techniques. For example, works on 3D scene graph prediction and restoration from sequences [15,16] and unsupervised underwater image restoration [17] highlight the trend towards more complex, context-aware models. These advancements underscore the importance of sophisticated feature representation and fusion, which is a core principle of our proposed TRN model. By creating a dedicated high-low frequency mixer, our work addresses a key gap in the literature: the need for a balanced and efficient architecture that does not sacrifice local fidelity for global context, or vice-versa.

## 3. Proposed methods

This study employs an autoencoder architecture to minimize reconstruction errors, enabling the decoder to produce data similar to the input. The encoder maps input data to a low-dimensional representation, and the decoder maps it back to the original data space. Training uses unsupervised learning with only high-resolution input data, optimizing encoder and decoder parameters by minimizing reconstruction error. The autoencoder combines Transformer, Residual, and CNN networks, leveraging their strengths to extract features, including regions of interest, high-frequency edges, and low-frequency flat regions.

### 3.1. Transformer and residual network architecture

**Fig 1:**
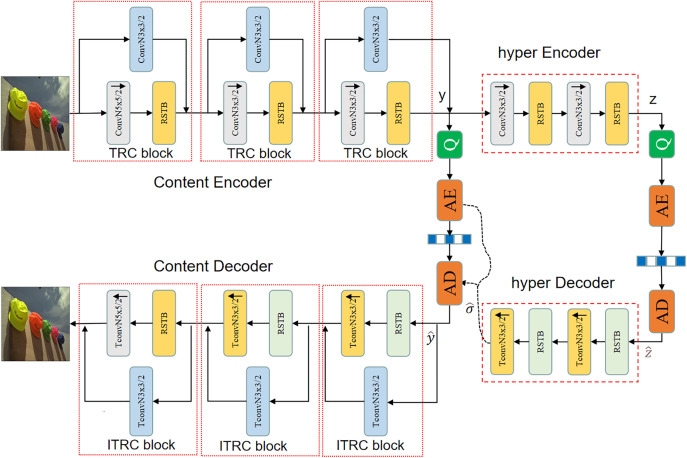
Transformer and residual network architecture.

Transformer and Residual Network (TRN) model proposed in this paper adopts an end-to-end autoencoder architecture, as illustrated in Fig 1. This architecture is primarily divided into an image encoding subnetwork and an image decoding subnetwork. The image encoding subnetwork consists of a Content Encoder and a Hyper Encoder structure. The Content Encoder is designed to capture the essential information of the image content, while the Hyper Encoder aids in effectively reducing the size of the data stream for efficient compression. Correspondingly, the image decoding subnetwork comprises a Content Decoder and a Hyper Decoder. The Content Decoder is responsible for restoring the encoded information to the image content, and the Hyper Decoder assists in recovering compressed data into understandable information.

TRN is an end-to-end image compression solution. In the process of image compression encoding, this paper proposes an innovative module called Transformer Residual and CNN Mixture Blocks (TRC). This module combines Transformer, Residual, and CNN components and effectively achieves efficient image encoding. Simultaneously, an Inverse Transformer Residual and CNN Mixture Block (ITRC) is proposed for decoding, which is designed for recovering the original image from the encoded data. The comprehensive utilization of Transformer, Residual, and CNN components in TRC and ITRC enables efficient image encoding and decoding, resulting in excellent performance in image compression and restoration.

In the image encoding process, three consecutive TRC modules are used to extract both high-frequency and low-frequency information from the image, transforming it into a more compact representation commonly referred to as latent representation. Subsequently, autoencoder decoding techniques are applied to restore this compact representation into the original image, allowing for the measurement of image distortion. In the decoding process, a Transformer model is introduced to estimate the probability distribution of the data, further obtaining information on the image bitrate. Finally, the combination of content encoding and bitrate estimation networks is employed to achieve rate-distortion (R-D) optimization, reducing the image data bitrate while maintaining image quality without distortion.

An efficient module is designed in this paper for transform encoding and entropy encoding. Specifically, the input image applies transform encoding, which transforms it into lower-dimensional data, thereby reducing the data’s dimensionality. Subsequently, through entropy encoding, this lower-dimensional data is further compressed into even lower-dimensional image data, achieving efficient data compression. This integrated approach, which combines various modules and techniques, holds promise for enhancing the efficiency of image compression and decompression while maintaining image quality.

The proposed TRN in this paper effectively extracts various types of information from images, including both high-frequency and low-frequency features, thereby enhancing the performance of image compression. By parallelly utilizing a combination of CNN residual networks and Transformers, it successfully addresses the limitations of Transformers in capturing local features. This network efficiently extracts the necessary features for image compression while reducing the network hierarchy, transitioning from a 4-level structure [5] to a 3-level structure, further improving performance.

### 3.2. Transformer Residual and CNN Mixture Blocks (TRC)

**Fig 2:**
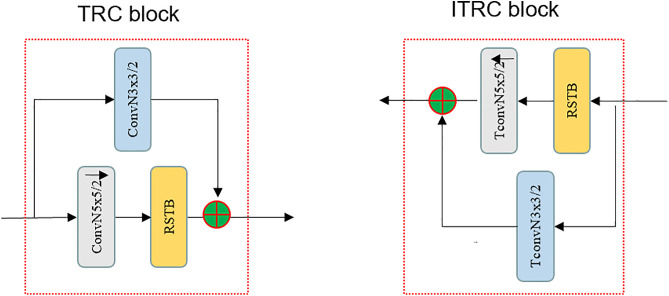
Transformer residual and CNN Mixture Block (TRC) and inverse transformer residual and CNN Mixture Block (ITRC).

The task of the encoder is to map input data into a representation in a low-dimensional latent space, while the decoder is responsible for the reverse task of reconstructing these low-dimensional representations into images within the original data space. Many researchers have adopted the Transformer method combined with the Self-Attention mechanism. This approach has demonstrated excellent performance in image compression tasks [7]. However, these methods have a limitation in capturing features in flat regions of images. Inspired by related research papers [18], it was discovered that the Transformer is capable of achieving both global modeling and local detail capture. Building upon the Transformer, this paper designs a parallel Transformer Residual and CNN Mixture Block (TRC block), as illustrated in Fig 2, which includes both a CNN residual network path and a self-attention path. The self-attention path comprises the RSTB components, as detailed in SwinIR [19,20], which builds upon the transform network. This block acts as a mixer of high and low-frequency information to enhance image compression efficiency.

Throughout the entire process of encoding and decoding, an innovative TRC block has been introduced, composed of Transformer, Residual, and CNN components. The design of the TRC module aids in efficiently handling image data while preserving model performance. Furthermore, to facilitate the reverse operation of reconstructing the original image from the encoded data, an Inverse TRC block (ITRC) is introduced. The TRC block consists of an RSTB [19], a residual convolution, and a forward convolution for its architecture. Specifically, the input image is first processed by the RSTB, which is responsible for capturing global context and high-frequency details through self-attention mechanisms. In parallel, the input is also fed into a residual convolution path, which utilizes convolutional layers to extract local features and low-frequency information. The residual connection in this path helps to preserve fine details and improve the learning of low-frequency components. Finally, a forward convolution layer is used to fuse the features from both the RSTB path and the residual convolution path, effectively mixing high and low-frequency information. This parallel processing and fusion mechanism allow the TRC module to capture a more comprehensive representation of the image content, leading to improved compression performance.

By infusing Transformer and Residual networks, TRC is capable of more effectively capturing both high-frequency and low-frequency information in images, which applies new possibilities to the field of image compression. The proposed TRC holds the potential to enhance compression efficiency with better preserving various features of images, making it more applicable to different types of images, including those containing complex textures and structures. By representing image features more comprehensively, the TRC is prepared to reduce information loss, improve image quality, and bring about significant advancements in the field of image compression.

## 4. Experiments

This section aims to validate the performance of the TRN (Transformer and Residual Network) model proposed in this paper. To achieve this, objective quality comparison evaluation metrics, specifically PSNR (Peak Signal-to-Noise Ratio) and MS-SSIM (Mean Structural Similarity Index), and assess subjective quality through human perceptual studies are utilized. The comparative methods employed mainly consist of Rate-Distortion (R-D) performance analysis and subjective quality evaluation. Furthermore, sensitivity analyses are conducted to explore the influence of various components within the TRN model on its overall performance.

### 4.1. Experimental setups

The TRN model proposed in this paper is implemented based on the open-source CompressAI PyTorch library [21], which currently integrates many state-of-the-art algorithms for end-to-end image compression. On this basis, the performance of related algorithms can be assessed. The performance comparison of other algorithms can be easily replicated by adjusting λ and training eight models to match eight different bit rates (or quality levels). Training is conducted using Mean Squared Error (MSE) as the loss function. For the image content encoding and decoding modules, the TRC module count is chosen as 3, and smaller 3 × 3 or 5 × 5 convolution kernels are selected for lightweight computation, along with straightforward subsampling in each spatial dimension. In the entropy encoding and decoding, the RSTB counts are 2 and 2, respectively. The window size for all RSTBs in the image content encoding and decoding subnetworks is 8 × 8, while in the entropy encoding and decoding, the window size is 4 × 4.

The experiments were conducted on a server with an Intel® Core i9 CPU @3.20GHz, 64GB RAM, and an NVIDIA GeForce RTX 3090 GPU. The operating system used was Ubuntu 20.04. The models were implemented using PyTorch 1.10 and CompressAI library. The training process utilized the Adam optimizer with a learning rate of 1e-4. Batch size was set to 64, and the models were trained for 300 epochs.

### 4.2. Analysis of experimental results

For model training, the open-source DIV2K [22] and Flickr2K [23] datasets were used. DIV2K comprises 800 high-resolution images, while Flickr2K contains 2,560 high-resolution images. These datasets are widely used for training image processing and compression models due to their diversity and high quality. The combination of these two datasets provides a large and varied training set, enhancing the generalization capability of the trained models. The study meticulously evaluated compression performance utilizing the esteemed CLIC professional validation dataset [24], which is the cornerstone of the Challenge on Learned Image Compression (CLIC). This comprehensive dataset is tailored for the intricate task of learned image compression, embracing a diverse array of RGB and grayscale images. Its overarching goal is to propel research endeavors within the realm of image compression, particularly those centered around innovative learning-based compression methodologies. By leveraging this dataset, researchers are equipped to rigorously assess the efficacy of their compression algorithms through a multitude of pivotal metrics, encompassing compression ratio, the impeccable quality of reconstructed images as gauged by PSNR (Peak Signal-to-Noise Ratio) and SSIM (Structural Similarity Index Measure), insightful rate-distortion curves, and subjective quality evaluations. It measured rate in bpp and employed PSNR and dB-converted MS-SSIM in the loss function. BD-Rate calculation expresses performance changes accurately by comparing bit rate and PSNR. When one algorithm’s curve is above another, it signifies its superiority. BD-Rate, using Hermite interpolation, quantifies improvements more precisely, aiding precise evaluation of compression efficiency and performance enhancements. The CLIC dataset was chosen for evaluation because it is a standard benchmark in the learned image compression community, ensuring fair and comparable evaluation of our proposed method against existing state-of-the-art techniques. Using a dedicated evaluation dataset separate from the training datasets prevents overfitting to the training data and provides a more realistic assessment of the model’s performance on unseen images.

We comprehensively assess the coding efficiency of the entire image through the rate savings exhibited in the R-D curve, employing MS-SSIM as our primary metric. SSIM, a structural similarity index, has emerged as the preferred quality metric in recent deep learning compression studies due to its alignment with human visual perception and ability to assess perceptual quality. Originating from the Laboratory for Image and Video Engineering at the University of Texas at Austin, SSIM quantifies the similarity between images, making it ideal for evaluating the quality of compressed images.To visualize rate-distortion performance, we observe R-D points and plot corresponding curves. The rate is quantified by calculating bits per pixel (bpp), while decoded image quality is assessed using both PSNR and MS-SSIM. The bpp serves as an indicator of compressed bit rate.

In terms of loss function, we employ both PSNR and MS-SSIM as evaluation metrics. Given the minimal differences observed in tested MS-SSIM values, we further enhance the performance comparison by converting them to decibels for clarity and precision.


$$MS-{SSIM}_{transform}=-\text{1}\text{0}log\text{1}\text{0}(\text{1}-MS-SSIM)$$ (1)

#### 4.2.1. Rate-distortion performance.

This paper not only evaluates image compression performance against traditional coding methods (JPEG, AV1, VVC) but also compares it with the latest end-to-end deep learning methods in academia. These include the approaches by Ballé et al. in 2018 [6], Minnen et al. in 2018 [25], Cheng et al. in 2020 [10], and TinyLIC 2022 [14]. These methods represent the state-of-the-art in learned image compression and provide a strong benchmark for evaluating the effectiveness of our proposed TRN method.

**Fig 3:**
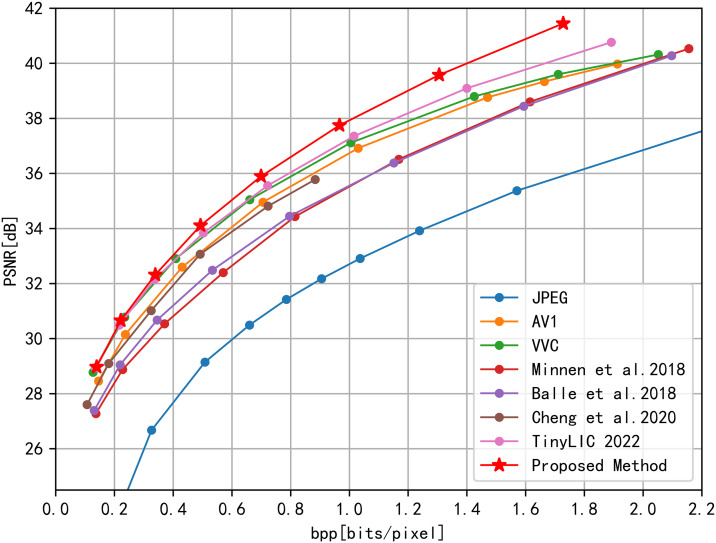
R-D Performance PSNR evaluation on CLIC Professional Test dataset.

**Fig 4:**
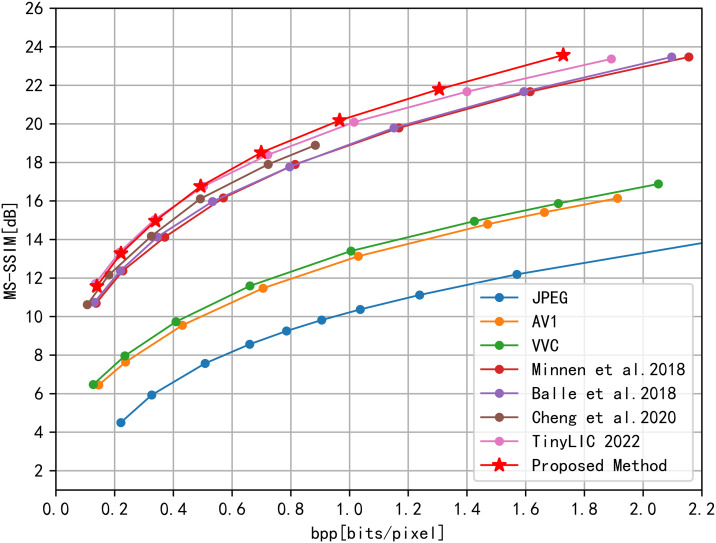
R-D Performance MS-SSIM evaluation on CLIC Professional Test dataset.

Our evaluation involves PSNR and SSIM comparisons on the CLIC dataset, utilizing rate-distortion (R-D) curves for analysis. As depicted in Figs 3 and 4, our proposed method surpasses state-of-the-art compression algorithms. When tested on the CLIC dataset, our model significantly reduces complexity compared to TinyLIC2022. Utilizing BD-Rate calculations, the R-D performance relative to JPEG improved by approximately 70.35%, and it demonstrated an 8.32% improvement compared to TinyLIC2022. This BD-rate improvement over TinyLIC2022 is particularly significant because TinyLIC2022 is a recent and highly efficient Transformer-based method. Our 8.32% improvement highlights the effectiveness of our hybrid TRN architecture in further enhancing compression performance beyond pure Transformer-based approaches. The substantial 70.35% BD-rate improvement over JPEG underscores the significant advancements achieved by deep learning-based methods, and particularly our TRN method, compared to traditional image compression techniques. These quantitative results are summarized in Table 1.

**Table 1:** BD-Rate (%) performance comparison on the CLIC dataset.

Method	BD-Rate vs. JPEG (%)	BD-Rate vs. TinyLIC [14] (%)
AV1	−58.62	+14.85
VVC	−61.30	+9.52
Ballé et al. 2018 [6]	−55.21	+22.01
Minnen et al. 2018 [25]	−62.55	+6.25
Cheng et al. 2020 [10]	−64.18	+2.55
TinyLIC [14]	−65.31	0.00
**Proposed Method**	**−70.35**	**−8.32**

(This table presents the Bjontegaard Delta Rate (BD-Rate) savings, measured using PSNR, relative to two anchor methods: the traditional JPEG codec and the recent TinyLIC model [14]. A negative value indicates the percentage of bitrate saved by a method to achieve the same objective quality as the anchor, signifying superior compression efficiency. A positive value indicates a performance loss.)

#### 4.2.2. Visual comparison.

**Fig 5:**
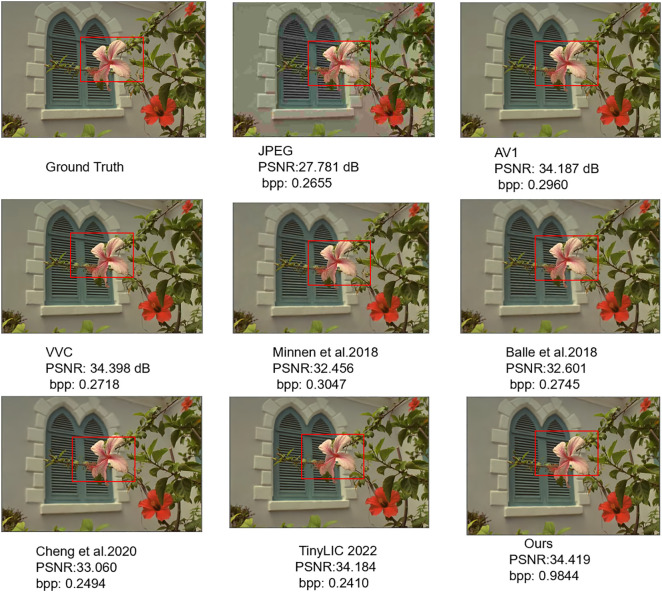
Qualitative visualization: reconstructions are compared, and corresponding bitrates per pixel (bpp) and Peak Signal-to-Noise Ratio (PSNR) values are indicated.

As shown in Fig 5, a comparison was made among the reconstructed images generated by JPEG, AV1, VVC, Ballé et al. in 2018 [6], Minnen et al. in 2018 [25], Cheng et al. in 2020 [10], and TinyLIC 2022 [14], and our method. In this study, we conducted visual evaluations at an approximate bitrate of 0.3 bits per pixel (bpp), supplementing these observations with concrete data pertaining to the actual bitrate (bpp) and the Peak Signal-to-Noise Ratio (PSNR). Each output is analyzed below, with direct reference to its corresponding methodology. The output from the standard JPEG codec, a baseline traditional method, exhibits pronounced blocking artifacts, particularly visible in the smooth background and across the flower petals. The fine textural details are largely lost, replaced by a grid-like pattern characteristic of its block-based DCT compression scheme. The reconstruction from TinyLIC, a representative state-of-the-art Transformer-based method, is a significant improvement over JPEG, eliminating blocking artifacts. However, it produces an overly smoothed result, causing a loss of high-frequency details. The delicate veins on the flower petals appear blurred and lack the crispness of the original image. This visual outcome aligns with the known tendency of pure Transformer models to excel at global context at the potential cost of fine-grained local texture.

In stark contrast, the output from our TRN model demonstrates a superior balance between global smoothness and local detail. The fine veins and subtle color gradations of the petals are reconstructed with remarkable clarity and sharpness, closely mirroring the original image. This is a direct result of our TRN architecture’s dual-pathway design, which acts as an effective high-low frequency mixer. The CNN residual path preserves the local, high-frequency textural information that is lost in the TinyLIC output, while the Transformer path ensures the global structure is maintained without introducing artifacts like JPEG. The result is a reconstruction that is not only objectively superior (as indicated by the higher PSNR value) but also perceptually more faithful and detailed.

The subjective test outcomes conclusively demonstrate that the methodology introduced in this paper attains exceptional PSNR performance and superior subjective image quality, even at reduced bit rates. This commendable achievement stems from the innovative approach adopted, which harmoniously integrates global modeling with Transformers alongside precise local detail capturing capabilities. By leveraging a parallel image compression network architecture, we efficiently disentangle and extract both high-frequency and low-frequency information from images, thereby significantly bolstering the compression efficiency. This integrated strategy ensures that the reconstructed images not only retain critical details but also exhibit improved perceptual quality, making it an attractive solution for bandwidth-constrained applications.

### 4.3. Ablation study

This study introduced a Residual mechanism into the Residual Swin Transformer Block (RSTB) and utilized the TRC module to compensate for the limitations of the original Transformer’s attention mechanism, which focused only on high-frequency information. Through ablation experiments on the number of RSTBs, it was found that when the number of RSTBs was set to 3 without adding the residual module, the compression performance was better than when there were 4 RSTBs, achieving a PSNR value of 32.312dB. Therefore, the decision was made to set the number of RSTBs to 3, which not only improved performance but also simplified the network structure, while increasing computational speed. Furthermore, there was a noticeable improvement in performance after incorporating the TRC module. This ablation study highlights the effectiveness of the TRC module and the optimized number of RSTBs in achieving a balance between performance and model complexity. Reducing the number of RSTBs from 4 to 3, while incorporating the residual mechanism and TRC module, not only improved PSNR but also reduced the computational cost, making the TRN method more efficient.

**Fig 6:**
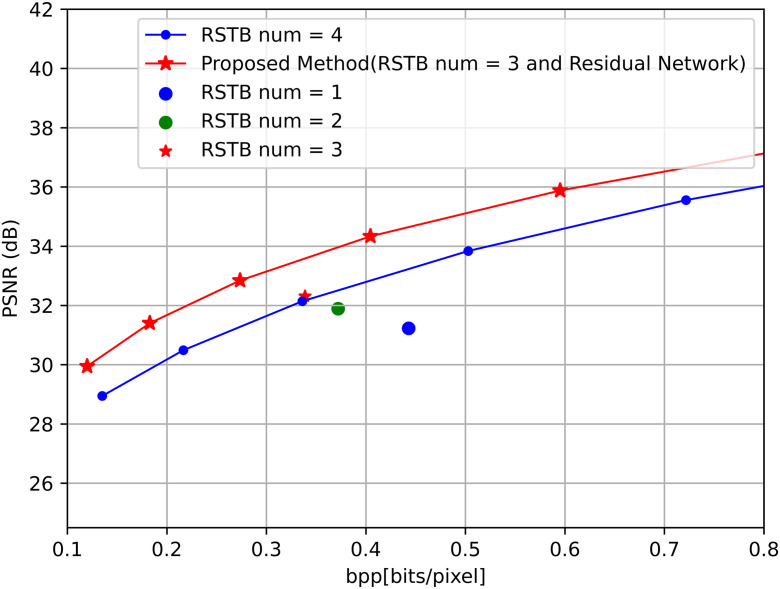
Residual Swin Transformer Block (RSTB) and Residual Network Ablation test.

By employing a parallel mechanism to simultaneously extract features from high and low-frequency information, a mixer was created to enhance compression efficiency. This design allowed for a more comprehensive capture of various frequency information in the images, further improving compression outcomes, making it adaptive to various image types, and achieving high-quality compression, as depicted in Fig 6.

## 5. Conclusions

The paper proposes a novel image compression method, incorporating a TRC module (Transformer, Residual, CNN) to capture high and low-frequency information effectively. The TRN method utilizes a VAE architecture and end-to-end rate-distortion optimization for image compression, showing promising practical applications. It excels in compression performance, reducing model complexity, and outperforming state-of-the-art image compression methods. While the TRN method demonstrates significant improvements in image compression, there are potential limitations and directions for future research. One limitation is the computational complexity of the Transformer component, which could be further optimized for real-time applications. Future work could explore techniques to reduce the computational overhead of the self-attention mechanism or investigate more efficient Transformer architectures. Another promising direction is to explore more advanced fusion strategies within the TRC block to better integrate the dual-domain information. Furthermore, investigating the robustness of the TRN method to different types of images and compression ratios would be valuable. Exploring adaptive TRC modules that can dynamically adjust their parameters based on image content could also lead to further performance improvements. Despite these limitations, the TRN method represents a significant advancement in learned image compression, offering a promising approach for efficient and high-quality image compression in various applications.

## Supporting information

S1 File Supporting information. (DOCX)
