# Supplementary material for: Effective image compression using transformer and residual network for balanced handling of high and low-frequency information
Source: PLoS One. 2025 Oct 3;20(10):e0333376. doi: 10.1371/journal.pone.0333376 (PMC12494236; doi:10.1371/journal.pone.0333376)
Supplement: S1 File — (DOCX) [file pone.0333376.s001.docx]

The datasets utilized in the manuscript comprise of open-source collections, specifically the DIV2K, Flickr2K, and CLIC datasets.

The DIV2K dataset can be accessed via the following URL: <https://data.vision.ee.ethz.ch/cvl/DIV2K/>.

Similarly, the Flickr2K dataset is available at <http://cv.snu.ac.kr/research/EDSR/Flickr2K.tar>.

For the CLIC2020 dataset, please refer to <https://data.vision.ee.ethz.ch/cvl/clic/professional_train_2020.zip>.

Additionally, these three datasets are also hosted on Baidu Netdisk at <https://pan.baidu.com/s/1QFr2eaeXnW7qooAqRak_yQ>, with the extraction code 'mvip' to facilitate access.
